# Clinico-morphological correlations in the categorization of holes between the ventricles

**DOI:** 10.4103/0974-2069.64367

**Published:** 2010

**Authors:** Brad A Friedman, Anthony Hlavacek, Karen Chessa, Girish S Shirali, Eowyn Corcrain, Diane Spicer, Robert H Anderson, Sinai Zyblewski

**Affiliations:** Division of Pediatric Cardiology, Medical University of South Carolina, Charleston, South Carolina, USA; 1 Division of Pathology, Medical University of South Carolina, Charleston, South Carolina, USA; 2 Division of Pediatric Cardiology, University of Florida, Gainesville, Florida, USA

**Keywords:** Doubly committed and juxta-arterial defects, interventricular communication, muscular defects, perimembranous defects, ventricular septal defects

## Abstract

Controversy still exists in the categorization of holes between the ventricles, although they are the most common congenital cardiac malformation. Advanced imaging techniques such as three-dimensional echocardiography and computed tomographic angiography offer superb anatomical details of these defects. In this review, we have sought to collate the features highlighted in different categorizations and identify their similarities, but also emphasize their differences. We hope that an analysis of this type, now achievable during life, using advanced imaging, might lead to the appearance of a unified system for diagnosis and description of holes between the ventricles.

## INTRODUCTION

As holes between the ventricles are the most frequent congenital cardiac malformations, it is surprising that so many issues regarding their categorization remain contentious. To some extent, this reflects the understandable desire to relate the morphology of the defects to the anatomy of the normal ventricular septum. Those with knowledge of the normal anatomy, however, will appreciate that some of the holes, notably those opening directly beneath the conjoined leaflets of the arterial valves, cannot exist in an otherwise normally constructed heart. This is because, in the normal heart, the free-standing infundibular sleeve lifts the leaflets of the pulmonary valve away from the base of the ventricular mass. Recognition of such relatively subtle features is crucial, if appropriate genetic counseling is to be provided to those whose children have deficient ventricular septation, as the holes opening directly beneath the leaflets of the arterial valves are more closely related in developmental terms to the common arterial trunk than to the more frequent defect bordered directly by the atrioventricular component of the membranous septum. These latter defects, nonetheless, have also been contentious, as there has been a debate as to whether they should be considered perimembranous, albeit this term is now so widely used that it is unlikely to disappear from the pediatric cardiological lexicon. The term itself, however, is not always used in the fashion in which it was initially defined.[1] In this review, we show how the use of newly developed diagnostic techniques, employing three-dimensional reconstruction, have revolutionized the recognition, during life, of the different types of holes that provide communication between the ventricles, as well as clarify the structure of the normal heart. We also hope to show how the use of the techniques can resolve the ongoing contentious issues concerning their categorization.

## EXISTING SYSTEMS FOR CATEGORIZATION OF HOLES BETWEEN THE VENTRICLES

Several popular, albeit disparate, approaches exist for the categorization of holes between the ventricles. A well-recognized approach[2] is to place the lesions in four groups, which are then labeled numerically. The defects placed in the first category occupy the area which, in the normal heart, is usually closed by the membranous part of the interventricular septum. Such defects have been described simply as membranous defects, albeit it was shown many years ago[3] that the area of septal deficiency is appreciably larger than the area of the interventricular component of the membranous part of the normal ventricular septum. Placed in the second group are the various holes with exclusively muscular borders that occupy various parts of the muscular ventricular septum. The third group is made up of holes bordered superiorly by the conjoined leaflets of the aortic and pulmonary valves, while the fourth group is made up of the so-called atrioventricular canal defects, although there is no universal agreement as to which holes should be placed in this latter category.[4]

In a second popular categorization,[5] the defects opening directly beneath the conjoined arterial valvular leaflets were interpreted as representing conal hypoplasia. In addition, the defects characterized by malalignment between the conal septum and the remainder of the muscular ventricular septum were grouped together as conoventricular defects.[5]

The third popular categorization emerged from Europe,[1] underscored by the concept that all holes, when viewed from the right ventricle, could be placed in one of the three groups, irrespective of how they opened into the different parts of the ventricle. It was this approach that spawned the use of the term perimembranous. These holes defined the presence of fibrous continuity between the leaflets of an arterial valve and an atrioventricular valve in their posteroinferior margin, or between the leaflets of the two atrioventricular valves, in the setting of a double outlet ventricle. Holes with exclusively muscular borders made up the second group recognized by the Europeans, while their third group contained the defects characterized by fibrous continuity between the leaflets of the arterial valves. This group included those bordered superiorly by the leaflets of a common truncal valve.[1]

When this latter categorization is reinforced with information as to the direction of the opening of the holes into the right ventricle, and the presence or absence of septal malalignment, then it is our belief that the expanded classification contains all the information provided by the other systems. In an attempt to understand some of the reasons for disagreement, nonetheless, we should define with precision the plane of space we consider to represent the defect, and to establish whether a hole described as an interventricular communication is the same as a ventricular septal defect.[4] As the holes self-evidently need to be related in morphological terms to the septum itself, we also show how the extent of the normal septum is clarified by the use of computed tomographic techniques. We recognize, of course, that this technique is not used, and should not be used, as the primary approach for diagnosis of different types of ventricular septal defects. All the computed tomographic images of normal anatomy shown in this review have been obtained from patients who have been investigated, to establish the precise origin of the coronary arteries and their course, relative to the arterial pedicles.

## THE ANATOMY OF THE NORMAL VENTRICULAR SEPTUM

**Figure 1:**
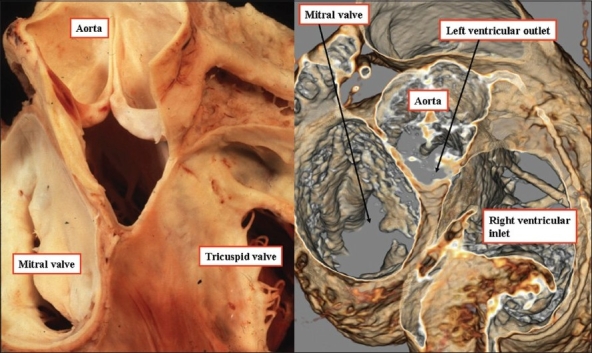
The heart, seen to the left hand, has been dissected by removing the atrial myocardium, along with the non-coronary sinus of the aortic valve. The dissection reveals how the subaortic outflow tract interposes between the mitral valvular orifice and the ventricular septum. The computed tomogram seen to the right hand and obtained from a patient suspected of having a coronary arterial anomaly, is prepared so as to replicate the anatomic dissection

**Figure 2:**
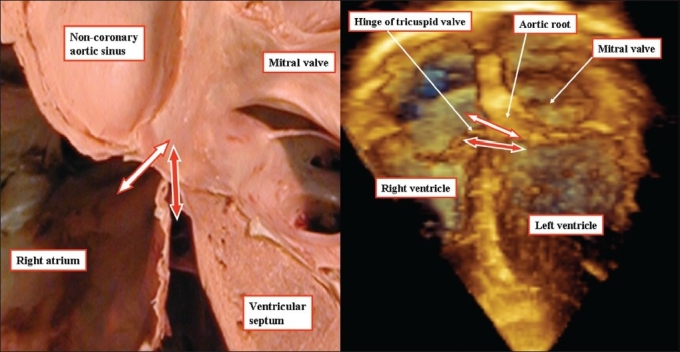
The heart shown to the left hand has been prepared by sectioning in the four chamber plane and removing the non-coronary leaflet of the aortic valve. The three-dimensional echocardiogram, seen to the right hand, replicates the anatomic preparation, showing how the hinge of the septal leaflet of the tricuspid valve divides the membranous septum into atrioventricular (white arrow) and interventricular (red arrow) components

**Figure 3:**
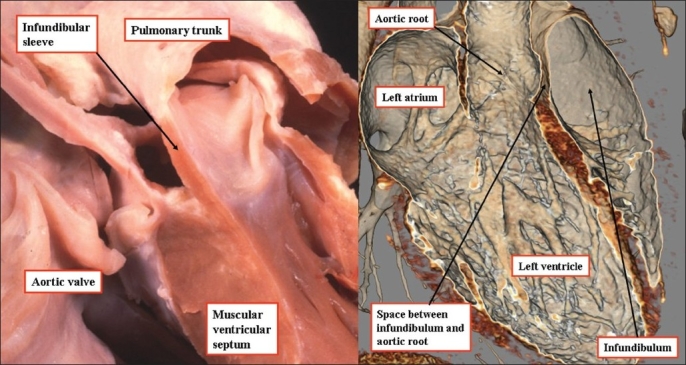
The dissection of the heart seen to the left hand was made by removing the ventricular septum interposed between the ventricular outflow tracts. It reveals how a sleeve of free-standing infundibular musculature lifts the leaflets of the pulmonary valve away from the base of the left ventricle. The computed tomogram, shown to the right hand, and performed to exclude a coronary arterial malformation, replicates the anatomic dissection

The normal ventricular septum has extensive muscular and very small fibrous components. The fibrous part is usually known as the membranous septum. The extensive muscular septum is not related, as might be expected, to the components of the ventricles themselves.[3] Goor and Lillehei[6] first pointed to the undoubted advantage of describing the ventricles in terms of their inlet, outlet, and apical trabecular components. Such a tripartite approach to the description does not suit the ventricular septum very well. This is because, in hearts with separate right and left atrioventricular junctions, very little of the normal muscular ventricular septum interposes between the ventricular inlets. In a normal heart, the subaortic outflow tract is interposed between the orifice of the mitral valve and the ventricular septum [Figure 1]. On account of this wedging of the subaortic area, the membranous septum interposes between the left ventricular outlet component and both the right-sided chambers, with the attachment of the hinge of the septal leaflet of the tricuspid valve dividing the fibrous septum into atrioventricular and interventricular components [Figure 2]. The larger part of the extensive muscular septum interposes between the apical ventricular components. There is virtually no muscular septum interposed between the ventricular outlets, the leaflets of the pulmonary valve being lifted away from the base of the ventricular mass by the free-standing infundibular sleeve [Figure 3].

**Figure 4:**
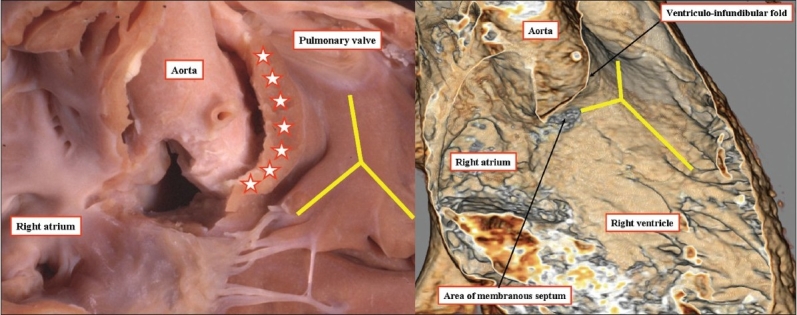
The anatomic dissection, seen to the left hand, was made by cutting away the parietal wall of the right ventricle and removing the area of the membranous septum, which occupied the interleaflet triangle between the non-coronary and right coronary sinuses of the aortic root. The computed tomogram, shown to the right hand, and performed to exclude a coronary arterial malformation, replicates the anatomic dissection. The yellow Y shows the location of the septomarginal trabeculation or septal band. The stars on the anatomic dissection indicate the location of the ventriculo-infundibular fold.

**Figure 5:**
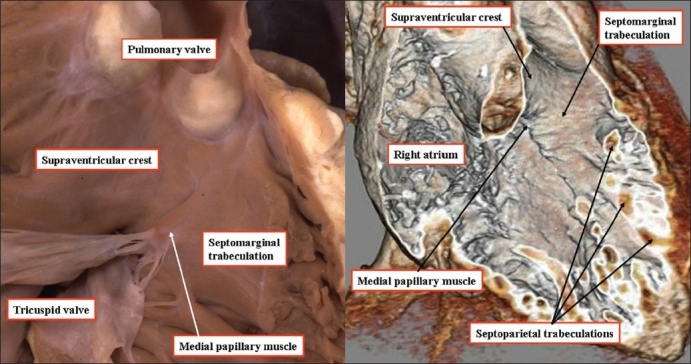
The heart shown to the left hand has been opened by a cut in its parietal wall and is photographed to show the supraventricular crest, which is the muscular wall interposed between the hinges of the tricuspid and pulmonary valves. As can be seen, the crest inserts into the ventricular septum between the limbs of the septomarginal trabeculation (See Figure 4). The computed tomogram, shown to the right hand and performed to exclude a coronary arterial malformation, replicates the anatomic dissection

The roof of the normal right ventricle is the so-called crista supraventricularis, or the supraventricular crest. Tomographic studies confirm dissections[7] showing that the greater part of the crest, which separates the leaflets of the tricuspid and pulmonary valves, is formed by the parietal wall of the right ventricle or the ventriculo-infundibular fold [Figure 4]. The distal part of the fold is continuous with the free-standing muscular infundibular sleeve. When viewed internally, the fold is seen to insert between the limbs of a prominent muscular trabeculation reinforcing the septal surface of the right ventricle,[7] known either as the septal band or the septomarginal trabeculation [Figure 5]. Located as it is on the septum, it cannot be supraventricular. It should not, therefore, be interpreted as representing part of the supraventricular crest. A small part of the crest inserting between the limbs of the septomarginal trabeculation usually also interposes between the subpulmonary and subaortic outlets. There are no obvious landmarks, however, showing where this potentially septal component stops, and where the free-standing infundibular sleeve begins. In the normal heart, therefore, it is better to simply describe the supraventricular crest, rather than seek to differentiate its component parts.[7] In the setting of many hearts with holes between the ventricles, in contrast, it becomes possible to recognize the components of the crest in their own right. The septal component of the crest can then be described as the outlet, conal, or infundibular septum. The ventriculo-infundibular fold can always be recognized because, as part of the inner heart curvature, it interposes between the leaflets of an arterial valve and those of an atrioventricular valve [Figure 4].[7]

## VENTRICULAR SEPTAL DEFECTS VERSUS INTERVENTRICULAR COMMUNICATIONS

**Figure 6:**
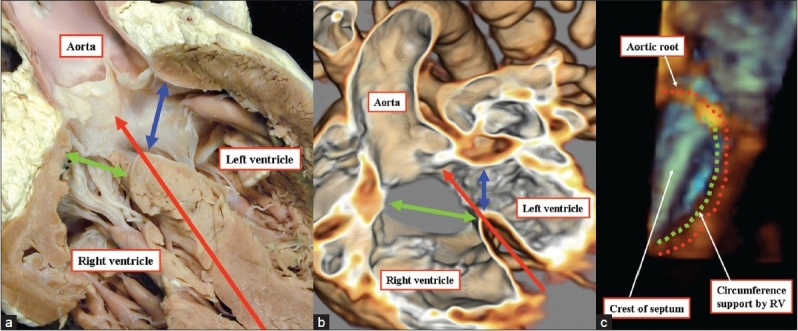
(a) The anatomic section shown to the left hand is a four-chamber cut through the overriding aortic valve in a patient with tetralogy of Fallot. The plane representing the interventricular communication is the upward continuation of the long axis of the ventricular septum (red arrow). It is the right ventricular margin of the cone of space subtended by the overriding valve (green double headed arrow) that is usually considered to represent the ventricular septal defect. The blue double-headed arrow is the effective outflow tract from the left ventricle. (b) A CT angiogram (middle panel) replicates the anatomic section. The plane representing the interventricular communication is the upward continuation of the long axis of the ventricular septum (red arrow). It is the right ventricular margin of the cone of space subtended by the overriding valve (green double headed arrow) that is usually considered to represent the ventricular septal defect. The blue double-headed arrow is the effective outflow tract from the left ventricle. (c) A three dimensional echocardiogram shown to the right hand shows that the plane of the ventricular septum truly divides the overriding aortic root into its right and left ventricular components, this plane being the interventricular communication

**Figure 7:**
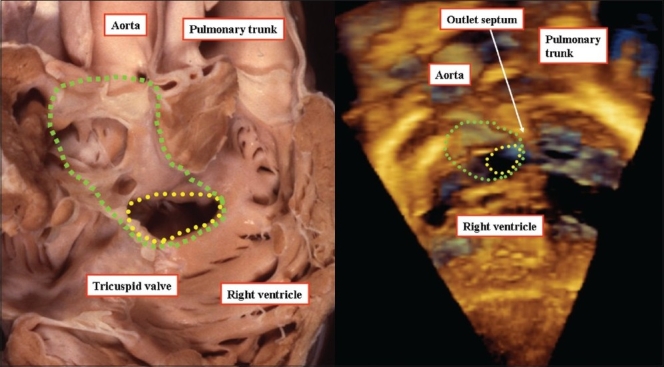
The anatomic specimen (left hand panel), and a three-dimensional echocardiogram from a different patient (right hand panel) show how, in a double outlet right ventricle, the interventricular communication is the effective outlet for the left ventricle (yellow dotted plane). Although often considered the 'ventricular septal defect,' it would be a disaster if this hole was closed surgically. Instead, the surgeon usually tunnels the interventricular communication to the aortic root (green dotted plane). It is this plane that should arguably be considered to represent the ventricular septal defect. Note that the muscular outlet septum is an exclusively right ventricular structure

In all the systems for categorization summarized in our introduction, as far as we are aware, the morphological features of the holes themselves have been defined on the basis of their margins as seen from the morphologic right ventricle. This approach itself is not without its potential problems, should the hole described as the ventricular septal defect also be considered to represent the same plane in space as the interventricular communication. In the presence of overriding of an arterial valve, for example, the interventricular communication represents the plane of space subtended between the crest of the ventricular septum, relative to the circumference of the arterial valve [Figure 6]. This plane is roofed by the closed leaflets of the overriding arterial valve during ventricular diastole. It cannot, therefore, be closed so as to restore septal integrity. Instead, it is the right ventricular margin of the space between the leaflets of the overriding valve and the crest of the muscular septum that is closed to restore septal integrity. It is this plane that is usually described by those using Anglo-Saxon languages as the ventricular septal defect. We presume that it is this hole, rather than the plane separating the cavities of the right and left ventricles, which is described as the interventricular communication by those using languages such as French, Italian, Spanish, and Portuguese. It is unclear, however, whether this usage also extends to the setting of the double outlet right ventricle. When both arterial trunks originate in their entirety from the right ventricle, it is the plane of space separating the cavities of the ventricles that represents the interventricular communication, functioning as the outlet for the morphologic left ventricle. This hole would obviously not be closed during those surgical repairs that restore biventricular communications. Rather, the interventricular communication is tunneled to one or other of the subarterial outlets. It is this interventricular communication, nonetheless, that is usually described as the ventricular septal defect by those describing patients with a double outlet right ventricle. If an analogy is made to the situation with overriding arterial valves [Figure 6], it would be the locus around which the surgeon places a patch from the crest of the muscular ventricular septum to the underside of the muscular outlet septum, itself exclusively a right ventricular structure, which should be considered to represent the ventricular septal defect [Figure 7].

## WHY ARE THERE DIFFERENCES IN THE DESPCRIPTION OF HOLES BETWEEN THE VENTRICLES?

When seeking to distinguish the various types of defects, those developing the various systems discussed a little earlier used different features of the holes for categorization. The time-honored numerical system, for example, used as its primary defining feature, the positions taken by the different holes relative to the components of the normal ventricular septum.[2] The system introducing the importance of the conus also concentrated on this feature,[3] but placed additional emphasis on malalignment and hypoplasia of the conal septum. The system proposed by the Europeans,[1] in contrast, used as its primary feature the structure of the borders of the hole as seen from the right ventricle, irrespective of the way the hole opened into the ventricle. The European group,[1] nonetheless, combined information on the direction of opening to the right ventricle with descriptions of the borders of the holes as seen from the right ventricle, so as to account for all the phenotypic variations, including malalignment of the septal structures. Recognition of such phenotypic variations, readily achieved using both cross-sectional and three-dimensional echocardiography, is crucial, as it is the knowledge of such variations that guides the surgeon to the location of the atrioventricular conduction axis.

## DIFFERENTIATING THE HOLES THAT OPEN TO THE INLET OF THE RIGHT VENTRICLE

**Figure 8:**
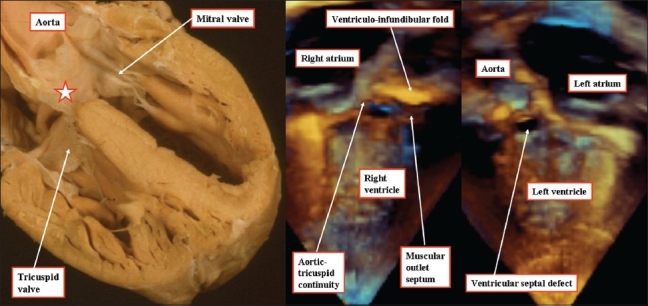
The anatomic section (left hand panel), and the three-dimensional echocardiograms from a different patient (right hand and middle panels), show that the defining feature of a perimembranous ventricular septal defect is the fibrous continuity in the posteroinferior quadrant of the hole between the leaflets of the aortic and tricuspid valves. Note that the atrioventricular component of the membranous septum (star) is an integral part of the area of fibrous continuity

There are four phenotypically discrete holes that open toward the right ventricular inlet. The most common type possesses, as part of its direct borders, fibrous continuity between the leaflets of the aortic, mitral, and tricuspid valves. This fibrous area incorporates the atrioventricular component of the membranous septum and, oftentimes, the interventricular component of the membranous septum can be recognized as a triangular fibrous flap that reinforces the posteroinferior margin [Figure 8]. When the Europeans produced their classification,[1] they described these defects as being perimembranous. This word was chosen for description because, when seen from the left ventricle, the hole was seen to extend around the components of the membranous septum that formed part of its perimeter. Others had considered holes of this type to be comparable to the septal deficiencies seen in atrioventricular canal malformations.[2,5] The major phenotypic feature of the atrioventricular canal defect, however, is the commonality of the atrioventricular junction.[8] The hole opening between the ventricular inlets in the setting of fibrous continuity between the leaflets of the aortic and mitral valves, in contrast, has separate right and left atrioventricular junctions, with the aortic outflow tract wedged between them [Figure 8]. Such holes, therefore, are not atrioventricular canal malformations in the sense that they possess a common atrioventricular junction. It may seem that the hole itself is directly comparable to the ventricular component of an atrioventricular septal defect. This is not the case. Another feature of atrioventricular canal malformations is the disproportion between the inlet and outlet dimensions of the ventricular septum, along with the unwedging of the subaortic outflow tract. In hearts with defects bordered by fibrous continuity between the aortic and mitral valves and separate atrioventricular junctions, the inlet and outlet dimensions of the septum are the same, the aorta being normally wedged between the mitral valve and the inferior component of the muscular ventricular septum. This means that the atrioventricular bundle is able to take its origin from an atrioventricular node, normally located at the apex of the triangle of Koch, with the bundle then extending posteroinferiorly relative to the hole, being seen to the right hand of the surgeon operating through the tricuspid valve.

**Figure 9:**
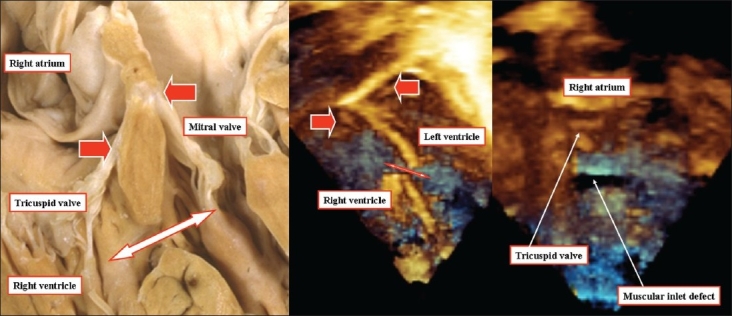
The anatomic section and the three-dimensional echocardiograms are from different patients with muscular defects opening to the inlet of the right ventricle (double headed arrows). The sections show the retained off-setting of the hinges of the mitral and tricuspid valves (red arrows). The right hand panel shows how, although the defect seems no more than a slit in the muscular septum, it has considerable length. This is one of the advantages of three-dimensional interrogation, as it shows the full extent of the septal deficiency

The second type of defect that opens to the inlet of the right ventricle has exclusively muscular borders. All agree that such holes are well described as being muscular inlet defects. They are distinguished by recognition of the persisting musculature of the septum between the normally offset leaflets of the mitral and tricuspid valves [Figure 9]. The presence of this superior muscular rim means that the conduction axis runs anterocephalad relative to the defect, being to the left hand of the surgeon operating through the tricuspid valve.

**Figure 10:**
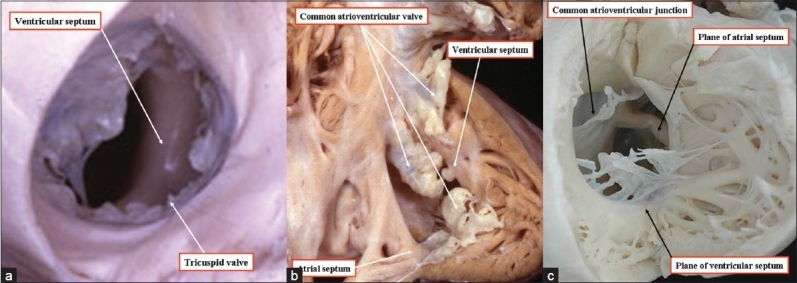
(a) The anatomic dissections show the fundamental difference between the inlet defect seen in the setting of the straddling tricuspid valve (left hand panel) and the atrioventricular septal defect with a common atrioventricular junction (middle panel). Although the muscular ventricular septum extends along the full length of the valvular orifice in both lesions, it is the right atrioventricular valve that overrides in the left hand panel, but a common atrioventricular valve in the right hand panel. There can, however, also be malalignment between the atrial septum and the ventricular septum in the setting of a common atrioventricular junction (right hand panel) (b,c) This also, nonetheless, is fundamentally different from the situation seen with the straddling and overriding of the tricuspid valve (left hand panel)

**Figure 11:**
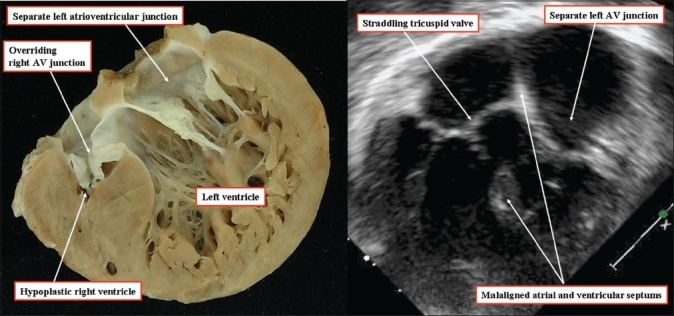
The anatomic section (left hand panel), and the echocardiogram (right hand panel) come from different patients. In both instances they show that the essence of straddling of the tricuspid valve is not in the malalignment between the atrial septum and the muscular ventricular septum, but in the setting of separate right and left atrioventricular junctions

The hole associated with straddling and overriding of the tricuspid valve also opens directly to the inlet of the right ventricle. Indeed, because of the overriding of the valvular orifice, the muscular ventricular septum in this setting extends across the full width of the right atrioventricular junction [Figure 10a]. This type of defect has also been considered to represent an atrioventricular canal type of ventricular septal defect.[9,10] In this the valvular orifice overrides the full length of the ventricular septum; there is a similarity between such defects and atrioventricular septal defects with a common atrioventricular junction [Figure 10b and c]. The crucial difference, however, is that the atrioventricular junction is common in the usual form of an atrioventricular septal defect [Figure 10b and c], but is the right atrioventricular junction when it is the tricuspid valve that is straddling [Figure 10a]. Furthermore, as with the hole having a fibrous posteroinferior border and opening to the right ventricular inlet [Figure 8], the hole associated with straddling of the tricuspid valve is found in the setting of separate right and left atrioventricular junctions [Figure 11]. Hearts containing such defects have none of the other phenotypic features of atrioventricular canal malformations. Additionally, in the setting of the straddling tricuspid valve, the atrioventricular bundle arises anomalously from a posteroinferior node, which does not take origin at its anticipated site within the apex of the triangle of Koch.[11] Failure to recognize this feature courts the danger of producing iatrogenic damage to the conduction axis during surgical repair.

**Figure 12:**
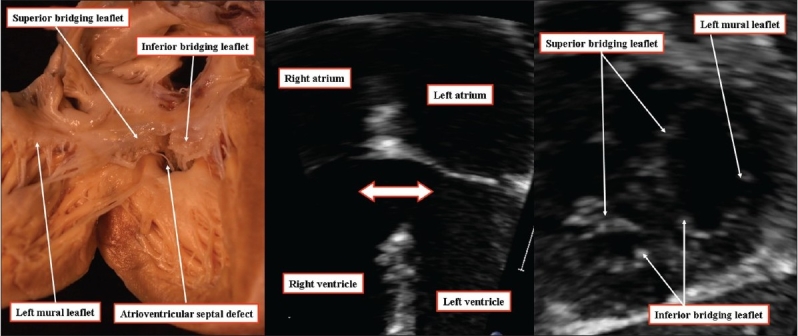
The anatomic image (left hand panel) and the echocardiograms (middle and right hand panels) are from different patients. They show how an atrioventricular septal defect with common atrioventricular junction can be arranged to permit only ventricular shunting when the bridging leaflets of the common atrioventricular valve are fused to the leading edge of the atrial septum. The right hand panel, in the short axis, confirms the trifoliate arrangement of the left atrioventricular valve

It is possible, of course, to find the true atrioventricular canal defect in which shunting is confined to the ventricular level. In these hearts, which possess all the phenotypic features of atrioventricular canal malformations, shunting is confined to the ventricular level because the bridging leaflets of the common atrioventricular valve are firmly attached to the superior margin of the atrioventricular septal defect [Figure 12]. The scooped-out ventricular septum inserts at the crux, with the atrioventricular conduction axis originating from a node, as expected for other types of atrioventricular septal defects, with a common atrioventricular junction.

## HOLES OPENING TO THE RIGHT VENTRICLE AT THE SITE OF THE MEMBRANOUS SEPTUM

**Figure 13:**
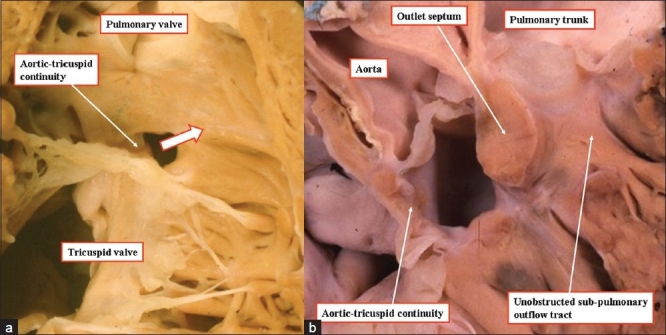
Anatomic images from different hearts show how perimembranous defects, defined because of fibrous continuity between the leaflets of the aortic and tricuspid valves, can extend to open into the outlet of the right ventricle (arrow) without (left hand panel) or with (right hand panel) malalignment of the muscular outlet septum

Overall, most patients with holes between the ventricles have muscular defects. In patients requiring therapeutic closure, in contrast, the most common type of defect opens into the right ventricle, in the area usually filled by the interventricular component of the membranous septum. As we have discussed, the hole occupies an area appreciably larger than the space usually filled by the interventricular component of the membranous septum,[2] and the membranous septum forms a direct border of the defect [Figure 8]. It is this type of hole that accounts for the most common congenital defect coded by those making surgical returns to the databases maintained by the Society of Thoracic Surgeons and the European Association of Cardiothoracic Surgeons.[14] The defect exists because of the deficiency of ventricular septal musculature in the environs of the hole usually closed by the membranous septum. It was for this reason that the European group[1] suggested that the hole be described as being perimembranous. The term is now firmly entrenched in the pediatric cardiac lexicon, and hence is unlikely to disappear. It is important to stress, therefore, that the criterion for description of any defect as being perimembranous is the presence of fibrous continuity between the leaflets of an arterial and atrioventricular valve, as part of the margin of the defect, as seen in the defect already described, which opens to the inlet of the right ventricle [Figure 8]. In all defects having this feature, apart from the ones associated with straddling and overriding of the tricuspid valve, the atrioventricular bundle will be related to the posteroinferior corner, and will be found to the right hand of the surgeon operating through the orifice of the tricuspid valve. This is the case whether such defects, which open directly beneath the inner heart curvature, extend so as to open more to the inlet [Figure 8], or open primarily to the outlet of the right ventricle [Figure 13].

## DEFECTS OPENING TO THE OUTLET OF THE RIGHT VENTRICLE

**Figure 14(a, b):**
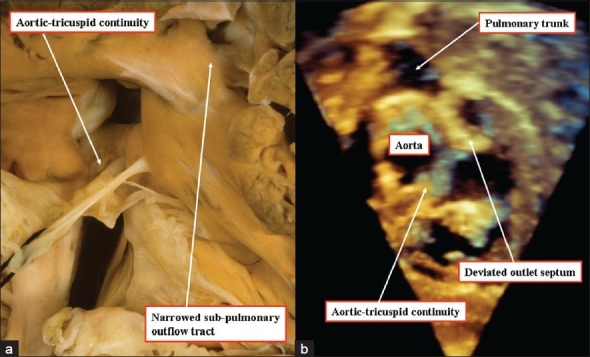
The anatomic image (left hand panel) and three-dimensional echocardiogram (right hand panel), taken from different patients, show how the ventricular septal defect is perimembranous in the majority of patients with tetralogy of Fallot, as fibrous continuity is present between the leaflets of the tricuspid and aortic valves. Subpulmonary stenosis is the consequence of anterocephalad malalignment of the muscular outlet septum

As with the defects opening primarily to the inlet of the right ventricle, defects with markedly different phenotypes can open in direct relationship to the ventricular outflow tracts. The most common of these is the hole that possesses, as part of its border, fibrous continuity between the leaflets of the aortic and tricuspid valves [Figure 13]. If the outlet septum is itself deficient, such holes can open directly beneath the subpulmonary infundibulum even in the absence of marked overriding of the right ventricular cavity by the leaflets of the aortic valve [Figure 13a]. However, quite often, such defects with a partly fibrous margin, described as being perimembranous in European categorization,[1] have associated malalignment between the muscular outlet septum and the remainder of the ventricular septum [Figure 13b]. In this setting, the defect opens to the right ventricle between the limbs of the septal band, however, in the absence of a subpulmonary infundibular obstruction. This is the so-called Eisenmenger defect.[15] In the past, such holes were recognized as being the ones producing pulmonary hypertension most easily. Nowadays, of course, the defect is closed on diagnosis, so as to prevent the progression of pulmonary vascular changes. Malalignment between the outlet septum, relative to the remainder of the muscular septum is also a major phenotypic feature of the conoventricular defect, typically seen in association with infundibular muscular stenosis, as in the tetralogy of Fallot [Figure 14a]. Irrespective of the presence or absence of subpulmonary stenosis, however, defects characterized by malalignment of the outlet septum can possess a different phenotype.

**Figure 15:**
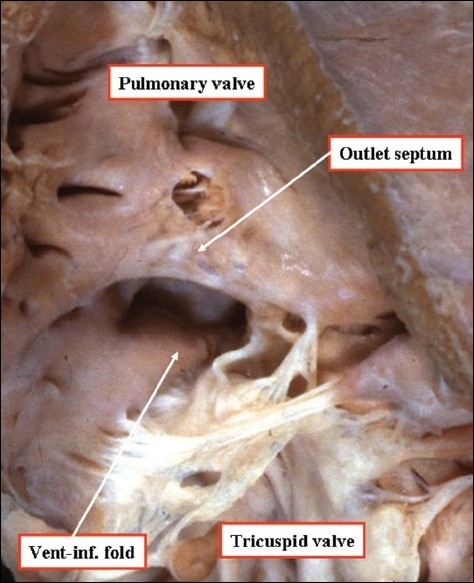
This anatomic image shows how defects with exclusively muscular borders can open to the outlet of the right ventricle

The second phenotypic defect opening beneath the ventricular outlets has exclusively muscular borders when viewed from its right ventricular aspect. The muscular border as seen from the right ventricle is the consequence of the fusion of the postero-caudal limb of the septomarginal trabeculation with the ventriculo-infundibular fold, the muscular bar thus formed interposing between the hinges of the leaflets of the aortic and tricuspid valves [Figure 14b]. Defects can also open to the right ventricular outlet with exclusively muscular rims in the absence of the malalignment between the muscular outlet septum, which is usually hypoplastic, and the remainder of the muscular ventricular septum [Figure 15].

**Figure 16:**
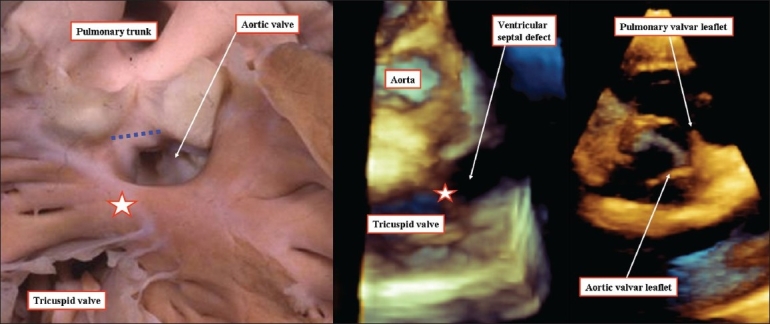
The anatomic image (left hand panel), and the corresponding three-dimensional echocardiograms (right hand and middle panels), taken from different patients, show that fibrous continuity between the leaflets of the aortic and pulmonary valves, due to failure of formation of the muscular subpulmonary infundibulum, is the cardinal feature of defects opening to the outlet of the right ventricle, but are directly beneath the arterial roots. Such defects are doubly committed and juxta-arterial. In the defects shown, the postero-caudal limb of the septal band fuses with the ventriculo-infundibular fold so as to produce muscular discontinuity (star) between the leaflets of the aortic and tricuspid valves

**Figure 17:**
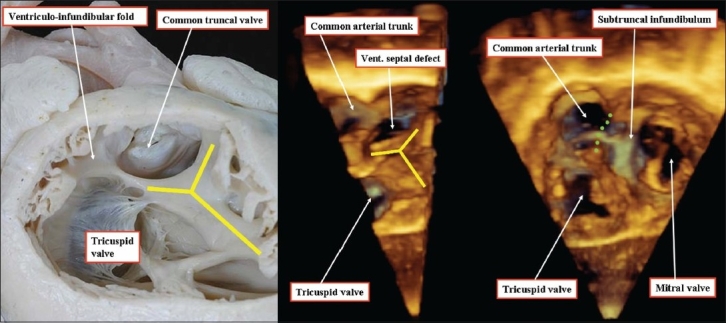
The anatomic image (left hand panel) and the three-dimensional echocardiograms (right hand and middle panels) show how the typical defect seen in the setting of the common arterial trunk is also directly juxta-arterial. In the images shown, there is again a muscular bar interposing postero-caudally between the leaflets of the truncal and tricuspid valves. The defect itself opens to the right ventricle between the limbs of the septomarginal trabeculation or septal band (yellow Y)

The third type of defect opening to the outlet of the right ventricle exhibits an entirely different phenotype. The defining feature of the holes of this type, is the fibrous continuity between the leaflets of the aortic and pulmonary valves [Figure 16]. They exist because of failure of formation of the muscular subpulmonary infundibulum, which normally lifts the leaflets of the pulmonary valve away from the base of the heart. The outlet septum itself can sometimes be visualized as a fibrous raphe beneath the conjoined leaflets of the arterial valves. Similar holes are found when there is a common arterial trunk exiting from the ventricular mass, but it is then a common arterial valve, rather than separate aortic and pulmonary valves, which guards the common ventriculo-arterial junction. In most defects of this type, which are doubly committed and juxta-arterial, a muscular rim is again formed posteroinferiorly by the fusion of the posterior limb of the septal band with the ventriculo-infundibular fold [Figure 17]. A minority of these holes, nonetheless, can extend so as to be bordered by a fibrous continuity between the leaflets of the arterial and atrioventricular valves, and hence also be perimembranous. When the defects are also perimembranous, the atrioventricular bundle is exposed in their fibrous posteroinferior margin.

## DIAGNOSTIC CONSIDERATIONS

Echocardiography, and specifically three-dimensional echocardiography, can now be used accurately to describe and categorize each type of hole permitting shunting between the ventricles. There should be no debate that it serves as the diagnostic modality best utilized to accomplish these goals. While other imaging modalities can also exquisitely define the nature of holes between the ventricles and show their relationships to the surrounding structures, these techniques have drawbacks not associated with three-dimensional echocardiography. For instance, when performed appropriately, gated computed tomographic angiography provides superb anatomic details, can be acquired quickly, and rarely requires sedation of the patient. Acquisition of such images, nonetheless, requires exposure to radiation. Although minimal, this exposure courts the known associated, inherent, long-term risk of this technique. Three-dimensional echocardiography also offers other advantages over computed tomography, such as higher temporal resolution and the ability to analyze the characteristics of the flow of blood. Magnetic resonance imaging can also reveal intracardiac structures, including holes between the ventricles, but the extensive time and expertise required to acquire and analyze the necessary images limits its practicality in these patients.

## CONCLUSIONS

In the past, it had been the examination of an autopsied specimen that had usually been taken as the gold standard for the understanding of a cardiac structure. Surprisingly, however, there has been a failure between those using autopsied hearts to reach consensus as how best to catagorize the holes between the ventricles. In part this reflects the difficulty, in autopsied hearts, of recognizing such subtle features as the presence of the sleeve of free-standing muscular subpulmonary infundibulum that lifts the pulmonary valvular leaflets away from the base of the ventricular mass. It is also intuitive to presume that the ventricular septum, like the ventricles themselves, possesses discrete inlet, apical trabecular, and outlet components. The ability now to dissect the heart during life, using cross-sectional or three-dimensional echocardiography illustrates that this is not the case.

Other reasons for the ongoing disagreements concerning the categorization of holes between the ventricles reflect the fact that different morphological features of malformed hearts have been chosen as the primary criterion for the developed heirarchy. On account of this, it has not thus far been possible to produce unity from mapped codes, as there is no certainty regarding equality between the phenotypes being mapped one to the other. In this review, therefore, we have sought to collate the features highlighted in different categorizations and identify their similarities, while also emphasizing their differences. We hope that an analysis of this type, now achievable during life, might lead to the appearance of a unified system for diagnosis and description of holes between the ventricles.
